# Sugars Replacement as a Strategy to Control the Formation of α-Dicarbonyl and Furanic Compounds during Cookie Processing

**DOI:** 10.3390/foods10092101

**Published:** 2021-09-05

**Authors:** Fabrizio Cincotta, Selina Brighina, Concetta Condurso, Elena Arena, Antonella Verzera, Biagio Fallico

**Affiliations:** 1 Department of Veterinary Sciences, University of Messina, Polo Universitario dell’Annunziata, 98168 Messina, Italy; fabrizio.cincotta@unime.it (F.C.); concetta.condurso@unime.it (C.C.); antonella.verzera@unime.it (A.V.); 2 Dipartimento di Agricoltura, Alimentazione, Ambiente (Di3A), University of Catania, Via Santa Sofia 98, 95123 Catania, Italy; sely81@hotmail.it (S.B.); bfallico@unict.it (B.F.)

**Keywords:** 3-deoxyglucosone, glyoxal, methylglyoxal, 5-hydroxymethylfurfural, Maillard reaction, furan, furan derivatives, headspace solid phase microextraction

## Abstract

In the last decade, several preventive strategies were considered to mitigate the chemical hazard accumulation in food products. This work aimed to study the effect of different sugars on the development of the main chemical hazard in cookies. For this purpose, model biscuits prepared using sucrose, fructose, and glucose were baked at different temperatures (150, 170, and 190 °C) and for different times (from 5 to 45 min), and the levels of α-dicarbonyl compounds, such as 3-deoxyglucosone (3-DG), glyoxal (GO) and methylglyoxal (MGO), 5-hydroxymethylfurfural (HMF), and furanic aromatic compounds were monitored. The replacement of sucrose in the cookie recipes with monosaccharides had as a consequence the highest accumulation of 3-DG (200–600 times higher), MGO, HMF, and furanic volatile compounds, while the use of sucrose allowed for maintaining the 3-DG, MGO, and HMF levels at less than 10 mg/kg dry matter in cookies for the estimated optimal baking time. Moreover, cookies with sucrose were characterised in terms of volatile compounds, mainly in terms of lipid oxidation products, while cookies with fructose or glucose baked at the highest temperature were characterised almost exclusively by Maillard reaction products, confirming a faster development of this reaction during baking at the studied temperatures.

## 1. Introduction

The Maillard reaction (MR) is an important and complex reaction that occurs in foods during thermal processing. It is responsible for the desirable aroma and colour development but also the undesirable chemical hazard formation.

In this regard, numerous studies have been undertaken about new chemical products that are formed during food processing due to safety concerns [1]. The main reported hazardous compounds are α-dicarbonyl compounds (DCs), furan, 5-hydroxymethylfurfural (HMF), acrylamide, and heterocyclic amines [2]. 

DCs are intermediate compounds that are formed in thermally processed foods during the early stage of an MR, caramelisation reactions and lipid oxidation [3,4,5,6,7,8]. It is well known that baked goods and, in particular, cookies are an important dietary source of DCs since they contain high concentrations of sugars, have a low moisture content and are subject to baked conditions. The high variability of 3-deoxyglucosone (3-DG), glyoxal (GO), and methylglyoxal (MGO) levels was reported in commercial cookies [8,9,10]. The formation and degradation of DCs are strongly related to the colour and aroma development in food, as they are important precursors of both brown and volatile aromatic compounds [8,11]. Furthermore, DCs showed antibacterial activity against several bacteria strains [12,13,14]. Besides the beneficial role of DCs in foods, several adverse effects were reported both in vitro and in vivo. Cytotoxic activity and cancer-promoting effects were attributed to DCs [8,15,16]. Moreover, it was reported that DCs pass almost unaltered through the in vitro gastrointestinal digestion phases and strongly depressed the microbial community [17]. Moreover, DCs play a role as reactive precursors of the advanced glycation end products (AGEs), and the accumulation of both DCs and AGEs was associated with several chronic degenerative diseases, such as diabetes mellitus, Alzheimer’s disease, cardiovascular disease, and atherosclerosis [18,19,20,21]. 

Furan and furan derivative compounds are formed in foods, mostly during heat processing, such as canning, cooking, baking, and roasting at 150 to 200 °C [22]. As such, they occur in several food items, such as coffee, baby foods, cereal, canned and jarred foods, meat, fish, and infant formula [23,24]. These compounds are derived from various natural precursors present in foods, including ascorbic acid, carbohydrates, amino acids, fatty acids, and carotenoids [23]. Furan was reported as being carcinogenic in animal models [25,26] and is considered “possibly carcinogenic to humans” [27,28,29]. The highest exposures to furan were estimated for infants, mainly from ready-to-eat meals. Grains and grain-based products contribute most to the highest exposure to furan for toddlers, other children, and adolescents, and it is also the second-largest contributor in all other age classes [25]. 

HMF is a furanic compound that is formed during thermal treatments of foods as an intermediate of the MR and under acidic conditions from the sugar dehydration reaction [30]. It is present in several types of heat-treated commercial foods and it is used as a quality parameter in various processed food [31,32,33,34,35,36,37]. In cereals and baking items, HMF was used to monitor heating processes [38] and could be considered as the most important chemical contaminants that occur in bakery products [39]. 

It was reported that HMF lacks significant genotoxic activity, but it can be converted in vitro and in vivo into 5-sulfoxymethylfurfural (SMF), which was recognised to induce a genotoxic and mutagenic effect in bacterial and human cells and promote colon cancer in rats [33,40,41]. 

In the last decade, several preventive strategies were considered to mitigate the chemical hazard accumulation in food products, such as changing the time/temperature profile of the heat treatment, varying the composition of the food matrix, and encapsulating the reactive compounds [2]. Removal interventions were also proposed [42,43].

This work aimed to study the effect of using different sugars and baking conditions on the main chemical hazard development in cookies. For this purpose, model biscuits that were prepared with different sugars, such as sucrose, fructose, and glucose, were baked for different times and temperature conditions, and the levels of 3-DG, GO, MGO, HMF, and aromatic volatiles, with particular attention to furanic compounds, were monitored.

## 2. Materials and Methods

### 2.1. Standards and Chemicals

High purity (*p* > 98%) 5-hydroxymethyl furfural (HMF), sucrose, fructose, glucose, o-phenylenediamine (OPD), glyoxal (GO) (40% in water), and methylglyoxal (MGO) (40% in water) were purchased from Sigma-Aldrich (St. Louis, MO, USA). 3-Deoxyglucosone (3-DG) (*p* > 95%) was purchased from Santa Cruz Biotechnology, Inc. (Santa Cruz, CA, USA). Methanol, acetic acid, and water were of HPLC grade and obtained from JT Baker (Deventer, Holland). Ethyl butanoate, 2-pentanone, hexanal, benzaldehyde, 1-hexanol, β-phenylethyl alcohol, limonene, 6-methyl-5-hepten-2-one, acetic acid, 2-methylpyrazine, furfural, acetyl furan, 5-methyl-furfural, and furfuryl alcohol were purchased from Merk (Milan, Italy).

Gemini NX C18 (150 mm × 4.6 mm, 5 μm) and Luna C18 (250 mm × 4.6 mm, 5 μm) columns were obtained from Phenomenex (Torrance, CA, USA). A CP-Wax-52-CB capillary column (60 m, 0.25 mm i.d.; coating thickness 0.25 μm) and Divinylbenzene/Carboxen/Polydimethylsiloxane (DVB/CARB/PDMS) SPME fiber were obtained from Agilent Technologies (Santa Clara, CA, USA).

### 2.2. Production of Cookies

All ingredients, except for glucose, were purchased from local supermarkets. Model biscuits were prepared according to a recipe described in the American Association of Cereal Chemists (AACC) Method 10–54 (American Association of Cereal Chemists (AACC) International, 2000) with some modifications. The recipe was as follows: 240 g of wheat flour, 100.8 g of sucrose or fructose or glucose, 96 g of butter, 3 g of salt, 2.4 g of sodium bicarbonate, 1.2 g of ammonium bicarbonate, and 52.8 mL of deionised water. 

According to the recipe, all the ingredients were thoroughly mixed using a dough mixer (model 1596, Ariete, Italy) for 7 min. Afterwards, the dough was laminated three times using a manual laminator (Imperia, Lusso, sp 150 model, Bologna, Italy) and was formed into discs with a diameter of 5 cm and a thickness of 0.2 cm. Seventy-five biscuits were produced from each batch of dough. Sucrose (SU)-, fructose (FR)-, and glucose (GL)-formulated cookies were baked in a laboratory oven (ThermoScientific, Herathermoven, Italy) at three different temperatures: 150 °C, 170 °C, and 190 °C for up to 25 min. The baking temperatures and times used in this study were set based on the cookies’ thickness and colour development and were in the range suggested for short doughs [44]. Cookies were sampled every 5 min and used for estimating the optimal baking time. The dough and cooking processes were carried out in duplicates.

### 2.3. Determination of the Estimated Optimal Baking Time (EOBT)

The evaluation of the optimal surface colour of the cookies samples was performed by 50 panellists (age 20–55 years; 22 males, 28 females), who were habitual cookie consumers. The panelists choose to participate in the research and signed the informed consent as our institution does not have an ethics committee for taste and food quality evaluation studies. The evaluation was conducted in a room at 25 °C with white sidewalls and uniform light. Each panelist received for each cookie formulation (SU, FR, and GL) and baking temperatures (150, 170, and 190 °C), a randomly ordered set of cookies obtained at different baking time (5, 10, 15, 20, 25 min). The panellists were asked to indicate for each set the preferred baked good based on the surface colour of the cookies. These samples were selected for chemical characterisations. Moreover, the EOBT (in min) was calculated as the weighted arithmetic mean from the answers given by the panellists. 

### 2.4. Determination of the Moisture Content, pH, and the Total Titratable Acidity

The moisture content was determined in triplicate using gravimetric analysis. The cookie samples were ground in a home grinder (La Moulinette, Moulinex, Écully, France, 2002), then an aliquot of milled sample was placed in an oven at 105 °C until the dry weight was constant [34].

The pH and the total titratable acidity (TTA) were measured on the ground samples according to [45] using a pH meter (Mettler Toledo, MP 220). The TTA results of the dry matter were expressed as millilitres of 0.1 M NaOH consumed to titrate to a final pH of 8.5, the suspension obtained by blending 10 g of sample with 90 mL distilled water.

All analyses were performed in triplicate, and the reported result of each analytical determination was the average of six values (2 sample replicates × 3 analytical replicates).

### 2.5. Dicarbonyl Compounds and HMF Extraction

An aliquot of the milled sample (1 g) (minced as described in Section 2.3) was transferred to a volumetric flask (10 mL) and 5 mL of deionised water was added. The solution was stirred for 10 min, then the sample was diluted to 10 mL with deionised water and centrifuged at 10 °C for 15 min at 8500 rpm (ALC 4128, Italy) [34]. An aliquot of the supernatant was filtered through a 0.45 μm filter (Albet) and directly analysed to determine HMF content, while dicarbonyl compounds were derivatised before the HPLC analysis. 

### 2.6. HMF Analysis

An aliquot of the filtered supernatant was injected into an HPLC system (Shimadzu Class VP LC-10ADvp) equipped with a DAD (Shimadzu SPD-M10Avp). A Gemini NX C18 column fitted with a guard cartridge packed with the same stationary phase was used. The HPLC conditions were as follows: 0.1% (*v*/*v*) acetic acid in water (94%) and methanol (6%); flow rate, 0.7 mL/min; injection volume, 20 μL. The wavelength range was 220–660 nm and the chromatograms were monitored at 283 nm [34].

HMF was identified by comparing the retention times and UV spectra from samples with a standard solution and by splitting each sample with the HMF standard. Quantification of HMF was performed using external calibration curves. All analyses were performed in duplicate, including the extraction procedure, and the reported HMF concentration was therefore the average of eight values (2 sample replicates × 2 extraction replicates × 2 HPLC replicates). The results were expressed as milligrams of HMF/kilogram of biscuit dry matter.

### 2.7. Determination of Dicarbonyl Compounds (3-DG, GO, and MGO)

An aliquot of the filtered supernatant was derivatised with a 0.6% OPD solution in water [46]. After 12 h, the derivatised mixture was injected into an HPLC (Spectra System) equipped with a diode array detector (UV6000LP) and an autosampler (AS3000) (Thermo Electron, San Jose, CA, USA). The HPLC column that was used was a Phenomenex Luna C18 and the HPLC conditions were as follows: eluent A was 0.1% (*v*/*v*) acetic acid in water and eluent B was methanol; the flow rate was 0.7 mL/min; the injection volume was 20 μL. The gradient program was: t0 85% A and 15% B, t10 65% A and 35% B, t15 35% A and 65% B, t25 100% B, and t30 85% A and 15% B. The detector wavelength was set to 312 nm [14]. All compounds were identified by comparing the retention times and UV spectra with those of standard solutions and by spiking each sample with standards (GO, Sigma Aldrich, 43612, 40% in water; MGO, Sigma, M0252, 40% in water; 3-DG; Santa Cruz Biotechnology, sc-220865, 10 mg, *p* > 95%). Quantification of each component was performed using external calibration curves. The extraction procedure and the analyses were performed in duplicate, the reported concentration of each dicarbonyl compound was therefore the average of eight values (2 sample replicates × 2 extraction replicates × 2 HPLC replicates). The results were expressed as milligrams of dicarbonyl compound/kilogram of biscuit dry matter.

### 2.8. Determination of Aromatic Compounds

The volatile aromatic compounds in the cookie samples were analysed at the EOBT using Headspace Solid Phase Microextraction Gas Chromatography–Mass Spectrometry (HS-SPME–GC–MS). An SPME fiber that was coated with Divinylbenzene/Carboxen/Polydimethylsiloxane (DVB/CARB/PDMS) was used for the extraction of volatile aromatic compounds. A total of 1.5 g of homogenised milled sample were inserted into a 7 mL glass vial and added with 2 mL of saturated aqueous NaCl solution. The sample was equilibrated for 15 min at 35 °C, thus the fiber was exposed to the headspace for 15 min. Afterwards, the fiber was withdrawn into the needle and transferred to the injection port of the GC at 260 °C and kept for 3 min for the thermal desorption of the analytes onto the capillary GC capillary column. 

The GC–MS analyses were performed using a Shimadzu GC 2010 Plus gas chromatograph that interfaced with a TQMS 8040 triple quadrupole mass spectrometer (Shimadzu, Milan, Italy) that was equipped with a CP-Wax-52-CB capillary column (60 m, 0.25 mm i.d.; coating thickness, 0.25 μm). The analysis conditions were as follows: injector temperature, 260 °C; injection mode, splitless; oven temperature, 50 °C held for 5 min, then increased to 190 °C at a rate of 3 °C/min, and to 240 °C at 6 °C/min; carrier gas, helium used at a constant pressure of 10 psi; transfer line temperature, 250 °C; acquisition range, 40–400 *m*/*z*; scan speed, 1250 amu/s.

Each compound was identified using mass spectral data, NIST’20 (NIST/EPA/NIH Mass Spectra Library, Wiley, Hoboken, NJ, USA) and the FFNSC 3.0 database, linear retention indices (LRI), literature data, and the injection of the available standards. LRIs were determined based on a homologous n-alkane hydrocarbon mixture and analysed under the same GC conditions; LRIs were calculated according to Van den Dool and Kratz equation [47].

The volatile compounds were quantified using the standard addition method. Stock solutions of individual standards were prepared by dissolving the appropriate amount of each compound in ethyl alcohol (95%) to obtain a final concentration of 0.2 mg/mL. The solutions were stored at under −30 °C. Furthermore, five different volumes of each stock solution were added to multiple aliquots of each sample. The sample alone was also analysed. Quantification was based on a calibration curve that was generated by plotting the detector response versus the amount spiked of each standard. The peak area of each compound was determined during three replicates and the average value was calculated.

To quantify compounds, the calibration curve of a compound of the same class of substances with the most similar chemical structure and retention time was used.

Each sample was analysed in triplicate.

### 2.9. Statistical Analysis

Data for each chemical parameter were submitted to an ANOVA (SPSS^®^ Statistics 13.0, Armonk, NY, USA) to evaluate the significant differences (*p* < 0.05) within samples baked at the same temperature and for the same time.

Microsoft XLstat software 2014 (Addinsoft, Paris, France) was used to perform the statistical analysis of the volatile data. ANOVA, Tukey’s test, and principal component analysis (PCA) were performed to understand the statistically significant differences and investigate the relationship between sugar formulations, baking conditions, and aroma volatiles. A heat map was generated to visualise the clustering of the multivariate data.

## 3. Results and Discussion

### 3.1. Effect of Sugars on Optimal Baking Time and Chemical Properties of Cookies 

Finding the optimal baking time (EOBT) for cookies is generally performed to achieve the desired surface colour [48,49]. Table 1 reports the EOBT at each temperature. At 150 °C, the EOBT was 21.3, 24.5, and 25 min for cookies prepared with glucose (GL), fructose (FR), and sucrose (SU), respectively. At 170 °C, the EOBT was about 20 min for all cookie formulations. At the higher baking temperatures, the differences between the EOBT values were lower. The increases in baking temperature from 150 to 170 °C and from 170 to 190 °C each took a progressive reduction of about 5 min off the EOBT for SU and FR cookies and 3–4 min for GL cookie samples. GL had the lowest EOBT due to a more intense surface colouration.

According to our EOBT trend, it was found that cookies formulated with sucrose needed more baking time to reach a similar browning level than cookies formulated with hexose [50].

Cookies that had a baking time near the EOBT were sampled and analysed to determine the moisture content, pH and TTA, HMF, dicarbonyl compounds, and aroma volatile compounds.

Sugar type, temperature, and time for the baking process had strong effects on the measured parameters (Table 2). 

The first difference was that the SU cookies had the lowest moisture content independent of the baking temperature and time (about 2% at 150–170 °C and 4% at 190 °C), suggesting a faster loss of water during baking. The FR samples baked at 150° and 170 °C had triple the moisture content (about 6%) relative to the SU cookies. The GL cookies baked at 150 °C had the highest moisture level (about 8%), probably due to the shorter baking time (20 min); when the GL cookies were baked at 170 °C, the moisture content was significantly different from the levels determined in the other cookies (Table 2). At the highest baking temperature, the GL and FR cookies had a similar moisture level. No significant differences were reported regarding the water loss between cookies produced with different types of sugars and cooked at the highest temperature (200–300 °C) [50].

Differences were also apparent for both pH and titratable acidity values (Table 2). Furthermore, in this case, the SU samples were significantly different from the other cookie samples independent of the baking temperature, recording the highest pH value and the lowest titratable acidity (Table 2). This trend was due to the lower reactivity of sucrose to the MR than monosaccharides. The FR and GL samples showed similar values for both pH and titratable acidity at different baking temperatures (Table 2). 

As concerns HMF and 1,2-dicarbonyl compound levels, significant differences were found between samples (Table 2). The levels of HMF were in a similar range (5–75 mg/kg) to that found in a survey of commercial cookies [7,51]. Generally, increasing the baking temperature increased the HMF levels in cookies. The HMF amount was lower in the cookies where sucrose was used as a sweetener in comparison with those containing fructose or glucose due to the necessary sucrose hydrolysis into monosaccharides before HMF formation [50,52]. In the SU samples, the HMF levels ranged from about 5.1 to 8.3 mg/kg in the samples cooked at 150 and 190 °C, respectively. It was reported that under an oven temperature of 300 °C, cookies with sucrose were associated with the lowest HMF accumulation rate, while at 300 °C, an inverse trend was found, in agreement with the drastic sucrose degradation [50]. The use of fructose brought about the highest levels of HMF, both at 150 and 170 °C (10.4 and 74.9 mg/kg, respectively), as the rate of fructose conversion into the first intermediate was the highest [52]. The GL cookies showed a different trend: at 150 °C, the samples had an HMF content that was similar to that found in the SU cookies (4.5 mg/kg), notwithstanding a shorter baking time; at 170 °C samples had an HMF content that was higher than the SU cookies and lower relative to the FR ones. These trends agreed with those reported by [50,53,54]. At the highest temperature, the cookies with monosaccharides showed similar HMF contents (about 22–23 mg/kg). The use of sucrose and the used baking temperatures allowed for maintaining the HMF level at less than 5 mg/kg dry matter in cookies at the EOBT. 

The substitution of sucrose with fructose or glucose drastically affected the production of the three DCs, 3-DG, GO, and MGO, particularly 3-DG (Table 2). The SU samples contained very low levels of 3-DG, ranging from 5.3 to 6.7 mg/kg. These amounts were lower relative to the range reported by [10] and similar to those reported for commercial cookies [7]. In cookies with monosaccharides (FR and GL), 3-DG was the major dicarbonyl and its concentrations ranged from 960.9 to 3517.9 mg/kg for the FR cookies baked at 190 °C and the GL cookies baked at 170 °C, respectively. The use of glucose or fructose brought about an accumulation of 3-DG that was as much as 200–600 times higher relative to the sample with sucrose. Higher concentrations of 3-DG and the other α-dicarbonyls were found in cookies containing fructose/glucose syrup [10]. Moreover, between the two monosaccharides, GL cookies had the highest levels of 3-DG, ranging from about 2113 to 3517 mg/kg dry matter. 

GO is a dicarbonyl compound that is mainly produced by the autoxidation of glucose [55], and MGO is formed by the retroaldolisation of the intermediate 3-deoxyglucosulose [56].

At the EOBT, the GO levels ranged from 10.3 to 16 mg/kg dry matter and no significant differences were found between the samples for all baking temperatures. A range of 4.8–20.5 mg/kg of GO was reported in commercial cookies and a lower level of GO was found in cookies that were prepared at the laboratory scale due to both the different baking processes and the type of sugar used in the formulation [9]. Moreover, in the SU cookies, the amount of GO was higher than of MGO, while in the FR and GL cookies, the MGO concentration was generally higher than for GO. This different behaviour was probably due to the different formation pathways: GO is formed via sugar oxidation during the heating process, unlike MGO, which is enhanced by the MR [9]. 

As concerns MGO, its level ranged from 8.3 to 39.8 mg/kg. The substitution in the recipe of sucrose with the monosaccharides led to an increase in MGO. In the SU cookies, MGO was about 8 mg/kg, independent of the baking temperature. In the GL cookies, the MGO amount was between 11.8 and 18.7 mg/kg. The MGO concentration was the highest in cookies with fructose, with a level ranging from 33.5 to 39.8 mg/kg dry matter (Table 2). Cookies with monosaccharides had the highest levels of HMF, 3-DG, and MGO, confirming that MGO was enhanced by the MR [9]. Different levels of MGO were found in commercial cookies, depending on the type of sugars used in the formulation, the baking temperature and time, and the presence of other ingredients [9,10]. Moreover, the lowest levels of MGO and acrylamide were found in cookies that contained ingredients such as sucrose, glucose, or sodium bicarbonate [9].

Thus, it is possible to produce cookies with the lowest levels of HMF and DCs but with the desired surface colour by using sucrose in the recipe and baking them at the optimal temperature and time.

### 3.2. Effect of Sugar Substitution on Aroma Volatile Compounds of Cookies

Table 3 reports the volatile compounds that were identified using HS-SPME–GC–MS in cookies from different sugar formulations at the EOBT, along with odour descriptors and their tentative origins. The analyses that were carried out allowed for identifying a large number of volatile aromatic compounds, of which, 37 had a signal-to-noise ratio higher than 10, and hence were quantifiable. Esters, ketones, aldehydes, alcohols, terpenes, C_13_-norisoprenoids, acids, pyrazines, and furanic compounds were identified. 

To obtain further information, a difference in means investigation using ANOVA and Tukey’s test was applied to the volatile compounds data (Table 3) and distinct trends were observed among the samples. 

As regards esters, ethyl-butanoate and ethyl-octanoate are usually associated with fruity notes and arise from lipid peroxidation (LO) and further esterification with ethanol by yeast and bacteria or enzymes that are already present among the ingredients [57]. In agreement with the above-reported data, the amounts of these compounds were not influenced by the sugar type used in the formulation and decreased as the temperature increased. 

Among the ketones, linear ketones, such as 2-pentanone, 2-heptanone, and 2-nonanone (sweet and fruity notes), arose from the LO [58,59]. Their concentrations were the highest in the GL and FR cookies, particularly when the 150 °C-20 min and 170 °C-20 min baking conditions were used. Their lower amounts at the highest temperature and shortest time (190 °C-15 min) could have been due to the increased content of the MR products, which, in addition to giving the characteristic bakery product aroma, were demonstrated to have antioxidant activity by protecting lipids from oxidation [59,60,61]. Diacetyl, arising from the MR, was also observed; it is responsible for buttery, sweety, and creamy notes and showed a content that was slightly higher in the GL and FR samples in the 170 °C-20 min and 190 °C-15 min baking conditions. Moreover, acetoin (sweet, buttery, creamy, dairy) showed the highest content in the FR and GL cookies, its content decreased as the cooking time and temperature decreased and increased, respectively. Acetoin was found in free gluten bread, and it could be enzymatically formed from pyruvate and could be converted into diacetyl non-enzymatically; it could also arise from the MR [57]. 4-Cyclopentene-1,3-dione, another MR product, showed statistically significant differences in FR and GL samples in the 190 °C-15 min baking condition; it was previously identified in model samples that were prepared with GL and hydrolyzed proteins subjected to the MR [62].

Aldehydes, in particular hexanal (green, fatty, leafy, fruity) and benzaldehyde (sweet, almond, fruity), are typical volatile compounds in bakery products [63] and are precursors of aromatic compounds, such as alcohols and esters. Of interest, the resulting content of hexanal, an LO product, was higher in the GL cookies at 150 °C-20 min baking condition. The same behaviour was also observed for the other aliphatic aldehydes (LO products), which can be volatile aromatic compounds that are already present in flour [64], and also for benzaldehyde, which can be formed from the amino acid phenylalanine via Streker degradation or oxidation [65].

Diacetone alcohol, an AGEs’ precursor that is produced during the MR, was the highest in cookies baked at 170 °C and 190 °C, particularly in the GL and FR ones. Huang et al. [66] showed that the formation of this compound is enhanced if alkaline conditions are present. β-Phenylethyl alcohol, an MR product that is responsible for a rose-honey-like odour and considered as a typical alcohol in bakery products, did not show any statistically significant difference among samples [67].

The acetic acid content was quite stable between the samples, even if a little increase in the FR cookies baked at 190 °C was observed. Acetic acid could be formed via fermentation or via sugar degradation throughout the MR [57,68]. 

Pyrazines are important aromatic compounds in bakery products, and they are compounds that arise from both MR and LO [69]. 2-methylpyrazine (roasted, burnt, sweet) did not show significant differences between samples.

Furanic compounds were, quantitatively, the main class of compounds identified in the cookie samples. They mainly arise from the MR but can also be produced during fermentation or from LO. In our samples, most of the furanic compounds showed statistically significant differences between samples. The content of 2-pentyl furan was higher in all formulations in the 150 °C-25 min and 170 °C-20 min baking conditions and lower in the samples baked at 190 °C-15 min. The content of dihydro-2-methyl-3(2 H)-furanone, also known as coffee furanone (woody, bready), was higher in the GL and FR cookies at 170 °C-20 min and 190 °C-15 min. It was also identified in brown sugar sponge cake as an MR product [59,70]. Furfural is formed from sugar dehydration during an MR. In the cookie samples, its content was the highest in all formulations in the 190 °C-15 min cooking conditions, but it also notably increased in the FR and GL cookies at 170 °C-20 min. It was identified in sponge cake cooked at 139 °C for 35 min and its content could increase if a protein source, such as leucine, was added [71]. Our results are in agreement with Srivastava et al. [71], who affirmed that the content of furfural in baked goods is higher if reducing sugars are present. Acetyl furan (bready, caramel) and 5-methyl furfural (sweet, caramel, bready) levels were found to be higher in all formulations in the 190 °C-15 min baking condition. 2(3H)-Furanone or γ-Butyrolactone (burnt, sweet, creamy) is a characteristic aromatic compound that is found in bakery products. Its content, as for 2-pentyl furan, was higher in the GL and FR samples at 170 °C-20 min and 190 °C-15 min baking conditions. Furfuryl alcohol, the product of furfural reduction, increased as the cooking temperature increased in GL cookies without showing differences in the SU and FR ones. 

**Figure 1:**
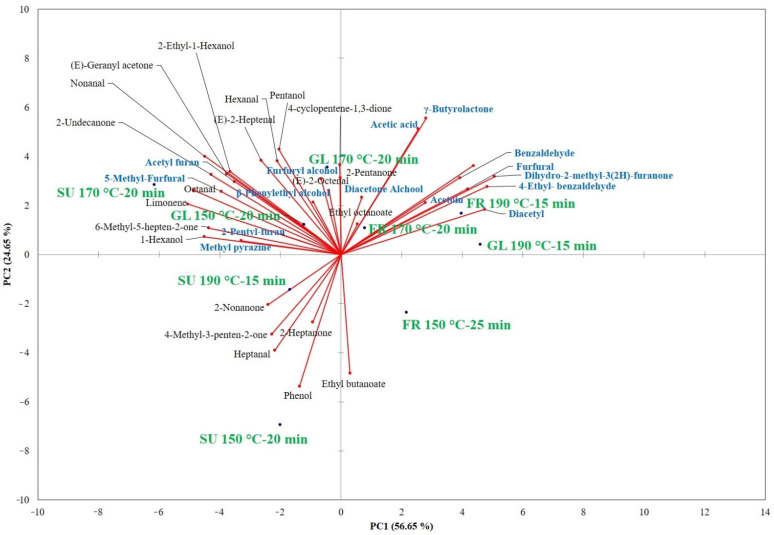
Principal component analysis (PCA) plot showing the multivariate variation between the SU, FR, and GL samples in terms of volatile aromatic compounds.

To better understand the influence of the sugar formulations and baking conditions on the volatile aromatic compounds, principal component analysis (PCA) was applied to all the compounds reported in Table 3 (Figure 1). 

The first two components together explained 81.28% of the total variance (PC1 56.64% and PC2 24.65%). PCA allowed for separating all the SU and the 150 °C-20 min GL cookies from the other samples since they were found on the negative side of PC1. These samples were found to be separated along the PC2 with the 190 °C-15 min and 150 °C-25 min SU samples on the negative side of PC2 and the 170 °C-20 min SU and 150 °C-20 min GL samples on the positive side of PC2. The 170 °C-20 min and 190 °C-15 min GL and FR cookies were well separated on the positive sides of PC1 and PC2, while the 150 °C-25 min FR samples were on the positive side of PC1 and negative side of PC2. The variables that were mainly correlated on the negative sides of PC1 and PC2 were 2-ketones, esters, and aldehydes; hence, LO products mainly characterised the SU cookies at 150 and 190 °C. Volatile compounds that are associated with LO (aldehydes, ketones, C_13_-norisoprenoids) and the MR (acetyl furan, furfuryl alcohol, β-phenylethyl alcohol, 5-methyl-furfural) were the variables that were mainly correlated on the positive PC2 and negative PC1 side, highlighting that reducing sugars were prone to undergo MRs faster than disaccharides.

The GL and FR cookies at 170 °C-20 min and 190 °C-15 min were located on the positive sides of PC1 and PC2, respectively. In this case, the variables that mainly influenced the separation were almost exclusively MR products (acetic acid, diacetyl, acetoin, benzaldehyde, 4-Ethyl-benzaldehyde, dihydro-2-methyl-3(2H)-furanone, Furfural, γ-Butyrolactone), demonstrating how this reaction took place more easily when reducing sugars were present and at a baking temperature of 170 °C or more. 

**Figure 2:**
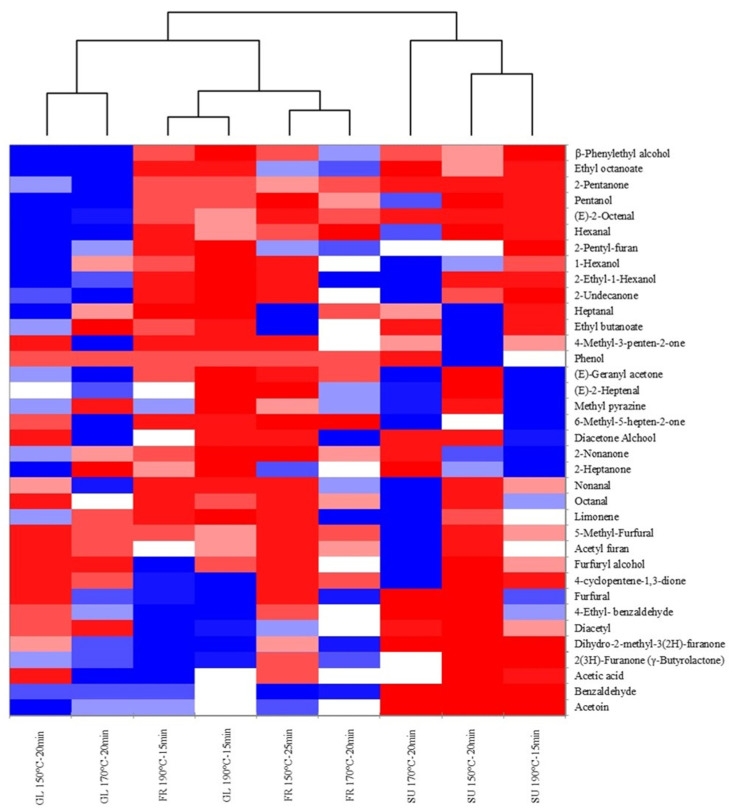
Heat map of the SU, FR, and GL samples using volatile aromatic compounds.

Figure 2 shows a heat map that gives a graphical view of the volatile compounds data. The heatmap colours of the matrix indicate the strength of the correlation between the samples and aromatic compounds. Dark blue indicates a negative correlation (−1), red indicates a positive correlation (+1), and the shade indicates an in-between correlation. As shown in Figure 2, two different clusters of samples formed: one containing SU samples and the other containing FR and GL samples. This second cluster was further divided into a cluster correlating GL samples at 150 °C-25 min and 170 °C-20 min and two subclusters, one containing GL and FR samples at 190 °C-15 min and other FR samples at 150 °C-25 min and 170 °C-20 min. These findings agree with the results produced using PCA.

## 4. Conclusions

In recent years, particular attention was focused on the chemical hazards formed in foods, particularly during thermal processing, and how to limit its accumulation. Cookies are one of the main commonly consumed ultra-processed foods, whose consumption was linked to the development of chronic diseases. This adverse effect is due to the poor nutritional quality of the food items and, lately, it was also hypothesised that a role is played by substances that are created during food processing.

The use of glucose and fructose syrup in cookie recipes is a widely used practice to improve the shelf life and cookies’ texture. The results of the present study showed that the replacement of sucrose in the cookies’ recipe with monosaccharides, such as fructose and glucose, had as a consequence the highest accumulation of α-dicarbonyl compounds (mainly 3-DG and MGO), HMF, and furanoic volatile compounds in cookies at the OBT. Moreover, the ability to retain water, the titratable acidity, the pH, and the main characteristic volatile aromatic compounds of the cookies were influenced. The use of both monosaccharides and the highest temperature produced volatile aromatic compounds that were almost exclusively characterised by MR products, indicating a faster development of this reaction during baking at the studied temperatures. 

## Figures and Tables

**Table 1:** Estimated optimal baking time (EOBT) in minutes of cookies produced using different sugars.

	SU Cookies			FR Cookies			GL Cookies		
Temperature	Time ^1^	Frequency	EOBT	Time ^1^	Frequency	EOBT	Time ^1^	Frequency	EOBT
150	25	100%	25	20 25	10% 90%	24.5	20 25	75% 25%	21.3
170	20	100%	20	15 20	10% 90%	19.5	15 20	30% 70%	18.5
190	15 20	90% 10%	15.5	15	100%	15	10 15	20% 80%	14

SU cookies: cookies formulated with sucrose; FR cookies: cookies formulated with fructose; GL cookies: cookies formulated with glucose. ^1^ minutes.

**Table 2:** Chemical parameters of cookie samples as a function of the type of sugar, baking time, and temperature.

Parameters	SU Cookies			FR Cookies			GL Cookies		
	150 °C 25 min	170 °C 20 min	190 °C 15 min	150 °C 25 min	170 °C 20 min	190 °C 15 min	150 °C 20 min	170 °C 20 min	190 °C 15 min
Moisture ^1^	2.0 ± 0.3c	2.3 ± 0.4c	4.2 ± 0.7b	6.1 ± 0.5b	6.2 ± 0.3a	6.3 ± 0.3a	7.9 ± 0.6a	4.9 ± 0.2b	5.5 ± 0.6a
pH	7.1 ± 0.02a	7.2 ± 0.08a	7.2 ± 0.04a	6.5 ± 0.02b	6.4 ± 0.12b	6.4 ± 0.10b	6.9 ± 0.13b	6.5 ± 0.16b	6.6 ± 0.09b
TTA ^2^	1.7 ± 0.1b	1.7 ± 0.1b	1.6 ± 0.1b	4.0 ± 0.0a	4.4 ± 0.4a	4.0 ± 0.3a	2.6 ± 0.1b	3.7 ± 0.1a	3.7 ± 0.1a
HMF ^3^	5.1 ± 0.9b	5.2 ± 1.5c	8.3± 0.4b	10.4 ± 1.6a	74.9 ± 5.3a	22.2 ± 7.1a	4.5 ± 1.0b	14.9 ± 1.9b	23.5 ± 3.2a
3-DG ^3^	6.7 ± 1.7c	6.1 ± 1.3c	5.3 ± 1.3c	1038.8 ± 133.0b	1162.3 ± 188.5b	960.9 ± 119.4b	2113.9 ± 384.6a	3517.9 ± 206.0a	3250.3 ± 54.7a
GO ^3^	10.7 ± 0.4b	10.3 ± 0.6a	10.7 ± 0.4a	14.5 ± 1.8a	16.0 ± 2.3a	14.1 ± 1.5a	14.3 ± 3.2a	15.4 ± 4.0a	15.6 ± 4.1a
MGO ^3^	8.4 ± 0.3c	8.3 ± 0.4c	8.40 ± 0.3c	33.5 ± 0.9a	39.8 ± 2.1a	37.5 ± 2.1a	11.8 ± 0.8b	17.3 ± 1.3b	18.7 ± 0.8b

SU cookies: cookies formulated with sucrose; FR cookies: cookies formulated with fructose; GL cookies: cookies formulated with glucose. ^1^ g/100g. ^2^ total titratable acidity expressed as mL NaOH N/10 on dry matter. ^3^ mg/kg dry matter. Data expressed as the mean ± standard deviation. Different letters in the same row indicate significant differences at *p* < 0.05 between cookie formulations with the respective temperatures and baking times.

**Table 3:** Volatile aromatic compounds (ng/g) in cookies at the EOBT using different sugar formulations.

				SU Cookies			FR Cookies			GL Cookies		
Compounds	LRI	Odour Description	Tentative Origin	150 °C 25 min	170 °C 20 min	190 °C 15 min	150 °C 25 min	170 °C 20 min	190 °C 15 min	150 °C 20 min	170 °C 20 min	190 °C 15 min
**Esters**												
Ethyl butanoate	1037	Fruity, sweet, apple	LO	11.72a	4.25c	3.54c	13.50a	6.38b	4.61c	7.45b	7.09b	3.89c
Ethyl octanoate	1437	Fruity, wine, waxy	LO	18.83a	12.78b	13.50b	23.10a	24.52a	16.34b	27.36a	19.19a	13.14b
**Total**				**30.55**	**17.03**	**17.04**	**36.60**	**30.90**	**20.95**	**34.81**	**26.28**	**17.03**
**Ketones**												
2-Pentanone	980	Sweet, fruity, banana	LO	17.76c	23.10c	21.67c	57.24b	51.55b	45.86bc	113.42a	80.00b	31.99c
Diacetyl	1062	Buttery, sweet, creamy	MR	nd c	2.47b	4.25b	6.38b	8.87a	12.43a	3.18b	2.12b	7.45a
2-Heptanone	1168	Fruity, spicy, sweet, herbal	LO	222.24b	245.36b	292.66a	235.05b	216.55b	192.02b	285.19a	129.07c	107.73c
Acetoin	1290	Sweet, buttery, creamy, dairy	MR	13.14c	10.65c	9.94c	37.32b	30.57b	32.34b	56.88a	33.77b	29.14b
2-Nonanone	1394	Roasty, cake crust, sweet	LO	92.09a	110.94a	112.00a	70.04b	82.84b	78.93b	87.11b	81.77b	68.26b
4-cyclopentene-1,3-dione	1598		MR	nd c	14.56b	10.65b	4.25b	13.85b	33.05a	7.45b	12.43b	40.52a
2-Undecanone	1605	Waxy, fruity, creamy, fatty	LO	19.19a	17.76a	15.27a	17.76a	20.96a	17.05a	23.10a	22.74a	14.92a
**Total**				**364.42**	**424.84**	**466.44**	**428.04**	**425.19**	**411.68**	**576.33**	**361.90**	**300.01**
**Aldheydes**												
Hexanal	1089	Green, fatty, leafy	LO	84.26b	86.75b	106.31b	112.36b	93.15b	99.20b	200.20a	195.93a	118.05b
Heptanal	1189	Fatty, oily	LO	81.77a	37.32b	22.03c	63.64a	33.41b	17.05c	65.06a	36.97b	16.70c
Octanal	1294	Aldehydic, waxy, citrus	LO	10.30b	11.72b	18.83a	7.45b	8.16b	8.52b	10.30b	9.58b	10.65b
(E)-2-Heptenal	1329	Pungent, green, vegetable	LO	2.47b	12.43a	15.27a	0.34b	6.74b	9.58a	8.87a	11.01a	4.25b
Nonanal	1400	Bready, cake crust	LO	33.05a	44.79a	55.10a	36.61a	38.74a	33.41a	56.53a	44.79a	40.88a
(E)-2-Octenal	1425	Damp, earthy	LO	6.38c	10.65c	9.23c	8.52c	11.36c	11.01c	65.42a	37.32b	18.83c
Benzaldehyde	1532	Sweet	MR/LO	18.12b	26.30b	28.43b	57.95a	56.17a	53.68a	52.26a	51.90a	44.08a
4-Ethyl-benzaldehyde	1729	Almond, sweet	MR	6.74b	7.45b	12.78a	10.30a	11.72a	15.63a	9.94a	12.78a	14.92a
**Total**				**243.09**	**237.41**	**267.98**	**297.17**	**259.45**	**248.08**	**468.58**	**400.28**	**268.36**
**Alcohols**												
Pentanol	1242	Fusel oil, sweet balsamic	LO	11.01b	12.07b	12.43b	11.01b	13.50b	13.14b	19.90a	17.05a	12.78b
1-Hexanol	1355	Ethereal, fusel oil, fruity	LO	12.43b	11.36b	10.65b	9.58b	11.72b	9.94b	17.76a	11.01b	5.32b
Diacetone alcohol	1368		MR	3.54d	85.69c	161.43b	22.39d	207.66a	205.89a	3.54d	213.71a	224.73a
2-Ethyl-1-Hexanol	1489	Citrus, fresh, floral, oily	LO	14.92a	14.56a	15.27a	15.63a	14.92a	14.21a	20.96a	19.90a	13.14a
β-Phenylethyl alcohol	1919	Rose, honey-like	MR	9.23b	7.81b	4.96c	7.81b	10.30b	8.52b	15.27a	14.21a	6.03b
Phenol	2003	Phenolic, plastic		8.52a	9.94a	11.36a	3.89b	6.03b	4.96b	4.61b	6.03b	5.32b
**Total**				**59.65**	**141.43**	**216.10**	**70.31**	**264.13**	**256.66**	**82.04**	**281.91**	**267.32**
**Terpenes**												
Limonene	1183	Citrus, lemon		10.30b	12.07a	12.78a	6.74c	8.16c	6.03c	14.21a	10.30b	5.67c
**C_13_-Norisoprenoids**												
4-Methyl-3-penten-2-one	1129	Musty, nutty chocolate		20.25a	7.09b	5.67b	1.05c	6.03b	9.58b	nd d	nd d	nd d
6-Methyl-5-hepten-2-one	1340	Fruity, musty apple		9.23b	13.85a	12.78a	4.25c	2.83c	5.32c	7.09b	7.81b	6.38b
(E)-Geranyl acetone	1862	Fresh, rose, leafy, floral		nd c	19.90a	23.81a	5.67b	7.81b	7.09b	14.21a	19.19a	nd c
**Total**				**29.48**	**40.84**	**42.26**	**10.97**	**16.67**	**21.99**	**21.3**	**27.00**	**6.38**
**Acids**												
Acetic acid	1464	Pungent, sour, vinegar	MR, C	1.40c	13.85b	12.43b	14.92b	17.41b	31.28a	12.07b	15.99b	19.54b
**Pyrazines**												
2-Methylpyrazine	1269	Roasted, burnt, sweet	MR/LO	2.47c	5.32b	7.09a	3.18b	4.25b	4.25b	4.25b	2.12c	nd d
**Furanoic compounds**												
2-Pentyl-furan	1221	Floral, fruity, green, earthy	F, LO, MR	56.00b	56.99b	22.30c	62.93b	56.49b	36.67c	94.65a	61.94b	27.75c
Dihydro-2-methyl-3(2H)-furanone	1264	Woody, bready	MR, CR	4.96c	6.38c	6.74c	13.50b	20.61a	21.67a	13.50b	18.83a	25.23a
Furfural	1468	Spicy, bready	MR, R	159.56c	167.59c	588.56a	291.02b	639.24a	651.28a	279.48b	616.66a	730.06a
Acetyl furan	1509	Bready, caramel	MR	0.53c	26.47b	43.94a	5.29c	35.47a	48.70a	7.94c	21.18b	33.35a
5-Methyl-Furfural	1584	Sweet, caramellic, bready	MR	10.04d	23.58c	47.17a	9.53d	36.63b	31.11b	6.52d	37.63b	55.69a
Dihydro-2(3H)-Furanone (γ-Butyrolactone)	1648	Burnt, sweet cream	F, MR	11.01c	18.47b	23.45b	29.85b	42.66a	47.28a	37.68a	40.88a	44.08a
Furfuryl alcohol	1666	Biscuit, cake crust, caramelised	LO, MR, CR	17.41d	102.85b	120.78b	74.37c	152.95b	310.13a	31.12d	53.80c	87.55b
**Total**				**259.51**	**402.33**	**852.94**	**486.49**	**984.05**	**1146.84**	**470.89**	**850.92**	**1003.71**

SU cookies: cookies formulated with sucrose; FR cookies: cookies formulated with fructose; GL cookies: cookies formulated with glucose. LO = lipid oxidation; MR = Maillard reaction; C = caramellisation; F = fermentation; different letters in the same row indicate a statistically significant differences (*p* < 0.05).

## Data Availability

Not applicable.
